# A highly sensitive plasma-based amyloid-β detection system through medium-changing and noise cancellation system for early diagnosis of the Alzheimer’s disease

**DOI:** 10.1038/s41598-017-09370-3

**Published:** 2017-08-21

**Authors:** Yong Kyoung Yoo, Jinsik Kim, Gangeun Kim, Young Soo Kim, Hye Yun Kim, Sejin Lee, Won Woo Cho, Seongsoo Kim, Sang-Myung Lee, Byung Chul Lee, Jeong Hoon Lee, Kyo Seon Hwang

**Affiliations:** 1 Department of Clinical Pharmacology and Therapeutics, College of Medicine, Kyung Hee University, Seoul, 02447 Korea; 2 Department of Electrical Engineering, Kwangwoon University, Seoul, 139-701 South Korea; 3 Department of Medical Biotechnology, College of Life Science and Biotechnology, Dongguk University, Seoul, Korea; 4 Center for BioMicrosystems, Korea Institute of Science and Technology (KIST), Seoul, 136-791 South Korea; 5 Department of Pharmacy & Integrated Science and Engineering Division, Yonsei University, Incheon, 21983 South Korea; 6 CANTIS.co, Sangnok-gu, Ansan-si, Gyeonggi-do, 426-901 South Korea; 7 Department of Chemical Engineering, Kangwon National University, Gangwon-do, 200-701 South Korea

## Abstract

We developed an interdigitated microelectrode (IME) sensor system for blood-based Alzheimer’s disease (AD) diagnosis based on impedimetric detection of amyloid-β (Aβ) protein, which is a representative candidate biomarker for AD. The IME sensing device was fabricated using a surface micromachining process. For highly sensitive detection of several tens to hundreds of picogram/mL of Aβ in blood, medium change from plasma to PBS buffer was utilized with signal cancellation and amplification processing (SCAP) system. The system demonstrated approximately 100-folds higher sensitivity according to the concentrations. A robust antibody-immobilization process was used for stability during medium change. Selectivity of the reaction due to the affinity of Aβ to the antibody and the sensitivity according to the concentration of Aβ were also demonstrated. Considering these basic characteristics of the IME sensor system, the medium change was optimized in relation to the absolute value of impedance change and differentiated impedance changes for real plasma based Aβ detection. Finally, the detection of Aβ levels in transgenic and wild-type mouse plasma samples was accomplished with the designed sensor system and the medium-changing method. The results confirmed the potential of this system to discriminate between patients and healthy controls, which would enable blood-based AD diagnosis.

## Introduction

The pathogenesis of Alzheimer’s disease (AD), the most common type of dementia, is potentially driven by excessive production and deposition of amyloid-β (Aβ) protein^1, 2^, and abnormal Aβ levels can be detected in bio-fluid such as cerebrospinal fluid (CSF) and blood^3–5^. The continuous monitoring Aβ levels could facilitate early AD diagnosis and treatment before the onset of AD symptoms^6–8^.

For that purpose, various types of biosensor utilizing electrochemical sensor^4, 5, 7, 9–11^, surface plasma resonance (SPR)^6, 12, 13^, field effect transistor (FET)^14^, and etc^15, 16^ have been reported to demonstrate the detection of Aβ. Among them, the electrochemical sensing methods have been most-practically developed to detect Aβ in CSF^4, 5, 10^ or interstitial fluid (ISF) of mice’s hippocampus^11^. However, the invasive collecting procedure for CSF have limitation to applicate in clinical status. The detection Aβ levels from blood could be the simplest and most powerful method to confirm the amount of Aβ without any crucial invasion which is needed to get CSF^17^; as the most widespread method for diagnosis of various diseases^6–8^.

The possibility of Aβ detection from blood was verified by the report of highly correlation of Aβ levels in CSF and blood^18^. Conventional Aβ detection methods, such as enzyme-linked immunosorbent assay (ELISA), do not have sufficient sensitivity or appropriate detection limits for AD diagnosis^19, 20^ on the basis of Aβ detection in the blood phase. The detection of Aβ protein, which is a strong candidate neuropathological biomarker for diagnosis and treatment of AD^21, 22^, requires excellent performance of sensor systems, due to the low concentration of Aβ in blood (dozens to several hundred picograms per milliliter).

In general blood based bio-sensing applications, extraction of plasma from whole blood by centrifugation or microfluidic device^23, 24^ is the most important key step for accurate and highly sensitive detection of biomarkers in blood. The use of plasma obtained from whole blood would enable much more sensitive detection of biomolecules^25^. For detection of low concentrations of biomolecules, sensor performance has also been enhanced through development of signal processing and auxiliary components^26, 27^, as same as the effort on preparation of plasma from whole blood. Although Aβ analysis has been widely performed using various sensor platforms, integrated systems with an electrical sensor platform and a signal processing and measurement system for practical blood based AD diagnosis using clinical samples have not been developed^7, 9, 28^.

In this study, we introduced a sensor system for blood-based Aβ impedimetric detection that involved combination of a signal processing system and medium changes (plasma to buffer) for AD diagnosis with high sensitivity. Interdigitated microelectrode (IME) sensors were used as an impedimetric sensor platform. The output signal processing system of the IME sensor was constructed for performance optimization and enhancement of signal interaction between recognition and target materials with cancellation and amplification functions for removal of parasitic capacitance, error, and noise. The hardness of Aβ detection in the plasma phase was also verified by analysis of the comparison between signal and differentiated signal. Analysis of the robust response of Aβ after the medium change with the microchannel of PDMS was performed using well-established antibody immobilization protocols. Using this approach, we could verify the response of Aβ in our designed sensing system and discriminate between Aβ protein precursor/presenilin 1 (APP/PS1) transgenic mice and wild type (normal) mice by measuring Aβ levels in plasma with high sensitivity and reproducibility to applicate in diagnosis of AD.

## Experimental

### Materials

Aβ protein fragment 1–42 (Aβ 42; Sigma-Aldrich Korea; amino acid sequence: DAEFRHDSGYEVHHQKLVFFAEDVGSNKGAIIGLM-VGGVVIA) was used in the experiments to develop and evaluate the AD diagnosis system. Monoclonal antibodies specific for Aβ 1-16 (6E10; Covance, CA, USA) were immobilized on the sensor surface for specific binding to Aβ protein fragment 1–42, as previously described^29^. Bovine serum albumin (BSA; Sigma-Aldrich Korea) was also immobilized for blocking nonspecific binding. Phosphate-buffered saline (PBS; pH 7.8, 1X; Corning) and 1% PBST solution (PBS with Tween 20; pH 7.8; Sigma-Aldrich Korea) were utilized as dilution media and washing materials, respectively.

### Fabrication of the IME and PDMS microfluidic channel chips

**Figure 1:**
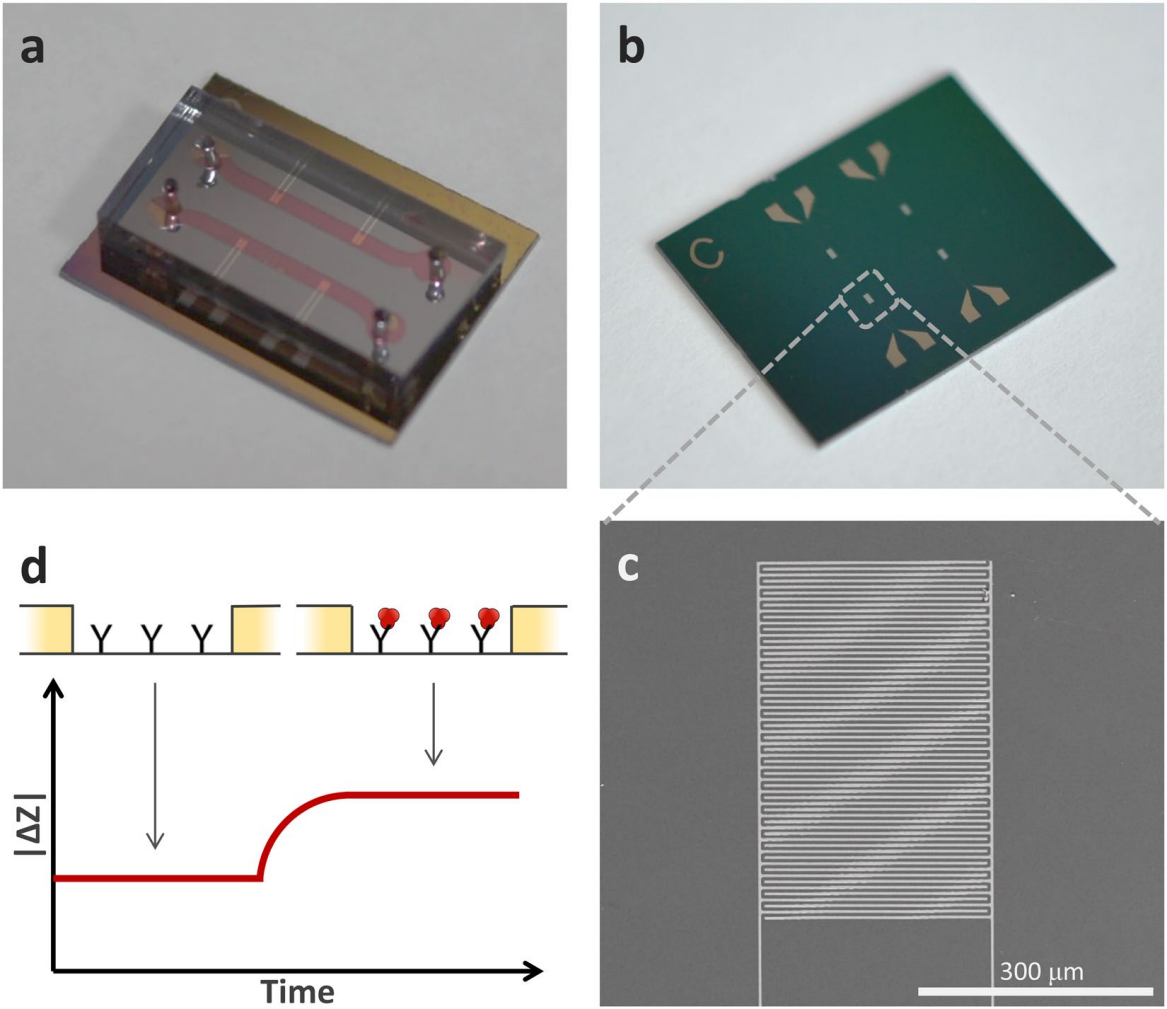
IME sensor scheme and sensing mechanism. (**a**) Medium-changeable microfluidic-channel-integrated device. (**b**) Image of the IME device. (**c**) SEM photograph of one IME pair. (**d**) Sensing mechanism for increase in impedance of the IME by interaction between Aβ and antibody.

The IME chip (as shown Fig. 1b) was combined with a microfluidic channel chip, as shown in Fig. 1a. The IME chip could detect biomolecules and the microfluidic channel chip could transport these to the sensing zone of the IME chips and be utilized as a chamber for media change. For the IME chip, a 300-nm-thick uniform silicon dioxide (SiO_2_) layer was grown on a Si wafer using thermal oxidation. The SiO_2_ layer, which is located between platinum (Pt) electrodes, was utilized as an insulator layer and biomolecule-immobilization surface. The Pt electrodes were patterned after the deposition of a 150-nm Pt layer. A 30-nm titanium (Ti) layer was deposited by sputtering as an adhesion layer between Pt and SiO_2_. The Pt electrodes were patterned by conventional photolithography and etched by ICP-RIE using an etcher (Oxford Instrument). As shown in Fig. 1c, a 5-µm-wide- and 300-nm-thick single Pt electrode was successfully obtained using this fabrication process. The 5-µm-wide gaps were also properly formed between all 30 pairs of electrodes. A polydimethylsiloxane (PDMS) microfluidic channel chip was also utilized to deliver the biomolecule-containing media to the sensing area of IME chips and easily change the media. The PDMS channel chip was fabricated using a conventional microelectrical and mechanical system (MEMS) fabrication method to obtain a 1-mm-wide and 50-µm-thick microfluidic channel. The target solution was injected into one channel and the buffer solution into another channel. Impedance of the fabricated IME chip was measured using commercial equipment (PGSTAT302N, Metrohm Autolab) for characterization. We determined that the coefficient of variation (C.V.) of fabricated IME chips-impedance was approximately 4.3%.

### Antibody immobilization for the recognition layer

**Figure 2:**
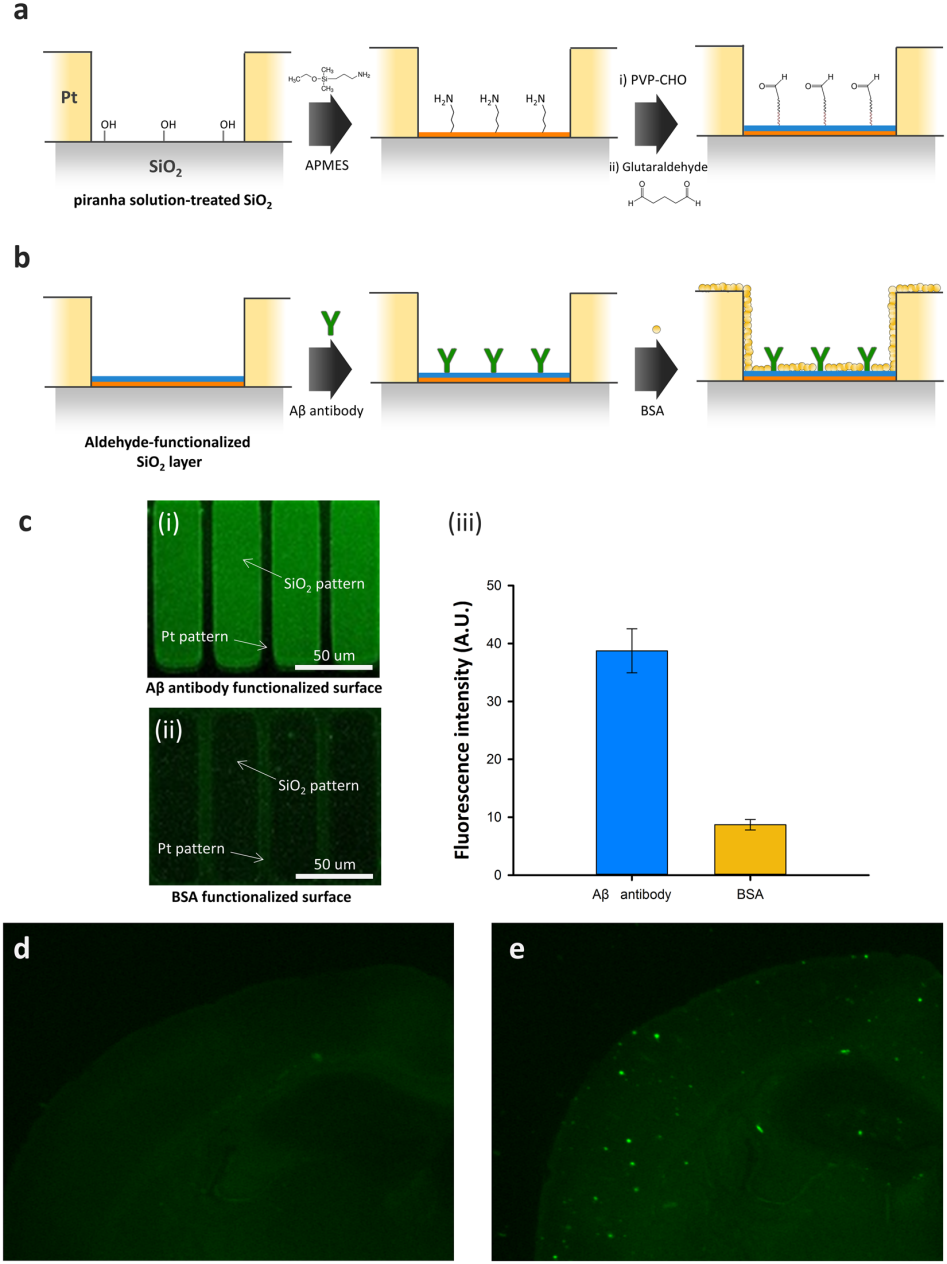
(**a**) Sequential process of surface modification. (**b**) Aβ antibody immobilization and BSA binding for blocking of nonspecific binding. (**c**) Aβ capture test using the quantitative analysis of the intensity of the fluorescent signal. (i) the fluorescent image of Aβ reaction on Aβ antibody functionalized surface. (ii) a negative control on the Aβ reaction with BSA functionalized surface. (iii) the graph of fluorescent intensity on Fig. 2a(i) and (ii). (**d**,**e**) The fluorescence images show Aβ plaque in the brain slices of wildtype (**d**) and transgenic (**e**) mouse.

The SiO_2_ surface on the IME chip was modified to immobilize the Aβ antibody for detection of Aβ. The surface modification processes are shown in Fig. 2. The processes have two steps: glutaraldehyde functionalization and antibody immobilization, as shown in Fig. 2a and b, respectively. First, the SiO_2_ surface was treated with a piranha solution (5:1 ratio of H_2_SO_4_ and H_2_O_2_) to remove contaminants for optimal formation of hydroxyl groups on the surface, as shown in Fig. 2a. The SiO_2_ surface was treated with a 3-aminopropyl triethoxysilane solution (APMES; 1% in isopropyl alcohol; Sigma-Aldrich) for 3 h for formation of an amine functional group on the surface. Next, the surface was washed with isopropyl alcohol, 100 mM NaHCO_3_ solution, and deionized water. The IME chip was dipped into polyvinyl pyrrolidone-aldehyde solution (PVP-CHO; 10 mM in 100 mM NaHCO_3_ solution; pH 9.0) for 6 h. The IME chip was then immersed in sodium borohydride (NaBH_4_; 10 mM in 100 mM NaHCO_3_ solution, pH 9.0; Sigma-Aldrich) for 1 h, followed by dipping into 1% glutaraldehyde solution for 1 h for formation of an antibody linker. After each treatment step with PVP-CHO, NaBH_4_, and glutaraldehyde solution, the IME chip was washed with 100 mM NaHCO_3_ solution and deionized water.

After the formation of the aldehyde group on the surface, the Aβ 1–16 (6E10) monoclonal antibody (10 µg mL^−1^ in PBS, pH 7.4) was immobilized with the formed aldehyde group for 1 h, as shown in Fig. 2b. Next, the other SiO_2_ surface and PDMS microfluidic channel chip were treated with BSA, applied for 1 h to block nonspecific binding, and washed with 1% PBST and PBS.

Fluorescein isothiocyanate (FITC, excitation and emission wavelength: 495 nm and 519 nm, respectively)-tagged Aβ and an SiO_2_ array with 30-μm width and 10-μm-wide Pt pitch were also utilized to verify the selective reactions of Aβ at the Aβ antibody layer, as shown in Fig. 2c. The upper figure, in which labeling with (i) is shown, displays the final fluorescent signals from the FITC-tagged Aβ reaction with immobilized Aβ antibody at the SiO_2_ substrate. The FITC-tagged Aβ was reacted with immobilized Aβ antibody that was prepared using the steps shown in Fig. 2a and b. The green color of fluorescent signals was measured only at the SiO_2_ patterns. For comparison as a negative control, the reaction of FITC-tagged Aβ at the BSA-functionalized surface was also performed as shown in Fig. 2c (ii) without Aβ antibody. Specific fluorescent signals were not measured from SiO_2_. The intensity of fluorescent signals at SiO_2_ was also quantitatively analyzed as shown in the graph of Fig. 2c (iii); 4.2-fold higher intense fluorescent signal was measured at the Aβ antibody layer containing substrate. Consequently, we could consider that our immobilization protocols for Aβ antibody are sufficient and robust for reaction with Aβ.

### Procedure of Aβ detection

In order to measure impedance change, we prepared the Aβ antibody functionalized IMEs chip for detection of Aβ. First, we measured impedance of IME in PBS buffer before reaction between Aβ and Aβ antibody. We injected Aβ samples into IMEs chip leading to interaction between Aβ and Aβ antibody, which was allowed to proceed for 20 min. IMEs chip was then washed with PBS buffer to remove non-reacted Aβ and other biomolecules. After washing out the IMEs chip, we measured impedance of IMEs, and analyzed sensing signal according to interaction with impedance change. And we utilized the normalized impedance change (|∆Z/Z_0_|) to compensate for the variation of electrical impedance between IMEs chips.

### Mouse sample preparation

#### Animals

Double-mutated APP/PS1 transgenic and wild-type mice were originally obtained from the Jackson Laboratory (USA; strain name: B6C3-Tg (APPswe, PSEN1dE9) 85Dbo/J; stock number 004462). Animals were maintained under a 12:12 h light/dark cycle with food and water available ad libitum at constant temperature and humidity. All animal experiments were carried out in accordance with the National Institutes of Health Guide for the Care and Use of Laboratory Animals (NIH Publications No. 8023, revised 1978) and all experimental protocols were approved by the Institutional Animal Care and Use Committee of Korea Institute of Science and Technology (KIST; Seoul, Korea), Yonsei University and Kyung Hee University (Seoul, Korea).

Mice were anesthetized with 2% avertin (0.02 mg/g. ip) and subjected for perfusion, performed with 0.9% saline followed by ice cold 4% paraformaldehyde (pH 7.4). Extracted brains were post-fixed for 18 hrs in 4% paraformaldehyde and immersed in 30# sucrose for 48 hrs for cryoprotection. These brains were cut 0.035 mm using a Cryostat (Microm HM 525, Thermo Scientific, Waltham, MA, USA) and mounted onto glass slides. Aβ plaques in brain tissue slides were visualized using Thioflavin S staining, as previously described^30^. We compared fluorescence images of brain slices between wild type (Fig. 2d) and transgenic (Fig. 2e) mouse prior to detect Aβ in mouse plasma. In the fluorescence image of transgenic mouse brain, the Aβ plaque were clearly observed. This brain-generated Aβ protein are flow into blood through the LRP1 channel^18^.

#### Plasma collection

Seven- to eight-month-old wild-type (female) and transgenic mice (female) mice (n = 9 per group) were anesthetized, and blood samples were obtained from the vena cava using a 26-gauge needle. Blood was collected in EDTA tubes (BD Vacutainer®, cat# 367835); the tubes were gently inverted 2–3 times, and the samples were centrifuged to separate cells and plasma (3,000 rpm, 15 min, 4 °C). A protease inhibitor cocktail (Roche cOmplete, Mini, EDTA-free) was added to the plasma samples, which were then analyzed immediately or stored at -80 °C until use, as previously described.

#### Peptide synthesis and purification

We synthesized Aβ 42 on Wang resin (0.25 mmol, 0.4 mmol g^−1^); 1.1 mmol of every amino acid, except the first amino acid, was synthesized by dimethyl sulfoxide (DMSO)-incorporated Fmoc solid phase peptide synthesis. For the first amino acid, alanine-42, 2.2 mmol was required for symmetric anhydride activation. The resin swelled in trifluoroacetic acid (TFA) for 2 min and was sequentially washed in dichloromethane (DCM) and dimethylformamide (DMF). N,N-diisopropylethylamine and DMF were added for 2 min to sequentially neutralize and wash DCM, and DMF. For symmetric anhydride activation, alanine-42 was dissolved in 1 mL each of DMF and DCM. Since the amino acid dissolved completely, N,Nʹ-diisopropylcarbodiimide was added, followed by approximately 5 min of shaking or sonication until the solution became unclear. The unclear solution and a catalyst, dimethylaminopyridine, were added to the swollen resin, followed by 1 h of shaking. The remaining amino acids were synthesized using a peptide synthesizer (CS336X, CSBio). The reagents used were as follows: as a coupling reagent, O-(benzotriazol-1-yl)-N,N,N′,N′-tetramethyluronium hexafluorophosphate; as a coupling base, N,N-diisopropylethylamine; and as a Fmoc deprotection reagent, 20% piperidine. We used 95% TFA to cleave peptides from the resin after synthesis. The peptide was thoroughly cleaved after approximately 2 h of shaking. The resin-filtered TFA was evaporated using a rotary evaporator. Cold anhydrous ether (at −20 °C) was added, and the mixture was centrifuged at 3,000 rpm for 10 min and then for 15 min. Finally, precipitated white peptide was segregated, dissolved in 50% acetonitrile, and lyophilized. Purification of crude Aβ 42 was performed using reverse-phase HPLC with a diphenyl column, as previously reported^31^.

## Results and Discussion

For Aβ detection in plasma samples, the basic characteristics of the designed sensing system, such as selectivity and sensitivity with synthetic Aβ, were verified. Enhancement of sensitivity with signal cancellation and amplification processing (SCAP; an additional signal processing) system and the medium-changing method was also confirmed. Finally, Aβ detection in plasma from AD (APP/PS1) transgenic and wild-type mice was accomplished with the designed method and system to show the possibility of Aβ detection in real plasma.

### Sensitivity and selectivity of the IME sensing system for Aβ with SCAP system

The response of IME sensing system according to the concentration of Aβ was measured to confirm the basic characteristics of sensor such as sensitivity and limit of detection with SCAP system. The Aβ was injected at concentrations ranging from 100 fg mL^−1^ to 1 ng mL^−1^ into each IME chip. Synthetic Aβ diluted with PBS buffer was utilized.

**Figure 3:**
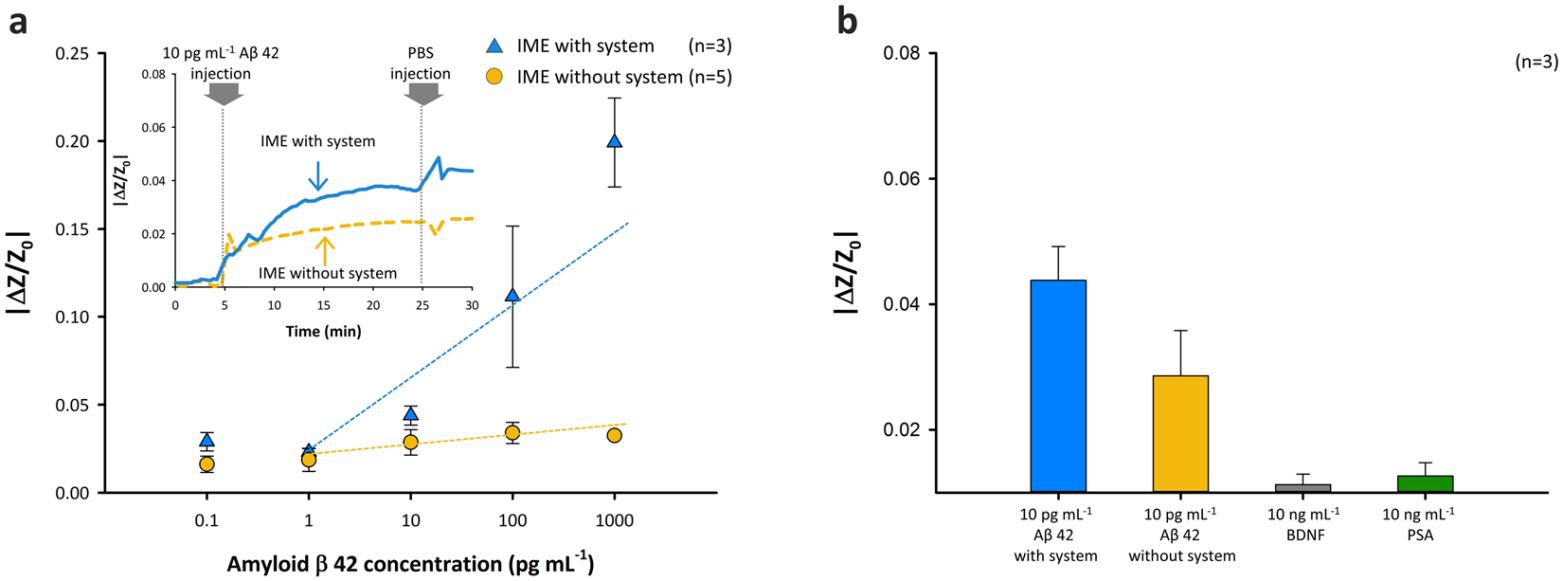
Sensing test for Aβ detection. (**a**) Sensitivity test of various Aβ concentrations and real-time detection by the IME with and without the designed system. (**b**) Selectivity tests using impedance changes for 10 ng mL^−1^ PSA, 10 ng mL^−1^ BDNF, and 10 pg mL^−1^ Aβ-injected IMEs.

As shown in the inset graph of Fig. 3a, the impedance changes according to the time flows were measured with and without SCAP system. The utilized frequency and concentration of Aβ were 100 Hz and 10 pg mL^−1^, respectively. The interaction of immobilized antibody and Aβ with the biosensor led to an increase in impedance, as previously described. Aβ (10 pg mL^−1^) was added after the first stabilization, as shown in the inset graph of Fig. 3a, both with and without SCAP system. The stabilization time was approximately 5 min after addition of the PBS buffer solution. When Aβ was injected into IMEs, we observed that impedance rapidly increased by approximately 2.0% after injection in the first 5 min and then became gradually saturated (approximately 2.6%) in the following 15 min (for a total measuring time of 30 min) when the designed SCAP system was not used. The reaction time for Aβ antibody–Aβ binding was also 20 min. The IMEs in the microfluidic channel were washed out with a PBS buffer solution to remove the remaining Aβ that did not react with antibodies. The impedance change was 2.6% for measurements obtained without SCAP system (dashed yellow line in the inset graph of Fig. 3a at 30 min) on injection of 10 pg mL^−1^ Aβ. Stabilization was also required after washing with PBS for the last 5 min. In contrast, the impedance of the IME with SCAP system (solid blue line in inset graph of Fig. 3a) dramatically increased and saturation was achieved at approximately 4.0% after 20 min, 15 min post-injection. After washing, the enhanced impedance change was 4.3% when the Aβ reaction was evaluated using SCAP system.

In the range of concentration from several picograms per milliliter to hundreds of picograms per milliliter, increases in the impedance of IMEs appeared to show a linear response according to Aβ concentration. Impedance changes with the Aβ solution were approximately 2.9%, 2.3%, 4.3%, 11.1%, and 19.9% for 100 fg mL^−1^, 1 pg ml^−1^, 10 pg mL^−1^, 100 pg mL^−1^, and 1 ng mL^−1^, respectively as shown in Fig. 3a. We also monitored the increase in the impedance of IME without the designed SCAP system. The increased values in IME impedance without SCAP system were approximately 1.6%, 1.8%, 2.8%, 3.4%, and 3.3% for 100 fg mL^−1^, 1 pg ml^−1^, 10 pg ml^−1^, 100 pg ml^−1^, and 1 ng mL^−1^, respectively. The increase in impedance due to the concentration of Aβ also appeared to have a linear association at concentrations ranging from 10 pg mL^−1^ to 1 ng mL^−1^. However, for the IME without SCAP system, the sensitivity and resolution for concentrations ranging from 10 pg mL^−1^ to 1 ng mL^−1^ were insufficient for discrimination of each Aβ concentration as shown in Fig. 3a. The gradients of slope according to the concentration obviously show the enhancement of sensitivity. The values of gradients were respectively 0.332 and 0.0035 (unit: 1/pg mL^−1^) with and without SCAP system. In case of utilizing SCAP system, approximately 100 folds steep slope was acquired. Although the variance in impedance change was broader than the signal from the measurement without SCAP system according to the Aβ concentration, the magnitude of the impedance signal was clearly higher when SCAP system was utilized. As shown in Fig. 3a, the increase in impedance was high enough to discriminate between protein concentrations ranging from dozens to several hundred picograms per milliliter with high sensitivity. The results tended to change according to the sensitivity and resolution for discrimination between AD patients and normal control samples for AD diagnosis. The limit of detection was hundreds of femtograms per millilitre.

To verify the selectivity, we monitored the impedance change of the IME with SCAP system after injecting a 1,000-times higher concentration of prostate-specific antigen (PSA; 10 ng mL^−1^) and brain-derived neurotrophic factor (BDNF; 10 ng mL^−1^) protein instead of 10 pg mL^−1^ Aβ into IMEs. PSA and BDNF proteins were injected into Aβ antibody–immobilized IME chips. Next, a PBS buffer solution was used for microfluidic channel washing. As shown in Fig. 3b, the increase in impedance with Aβ injection at the IMEs was higher than that associated with the changes observed using 1 ng mL^−1^ PSA and BDNF injections; about 1.1% and 1.2% impedance changes, respectively, were determined. We confirmed the selective detection of Aβ in IME chips.

### Compatibility of medium change for plasma samples

**Figure 4:**
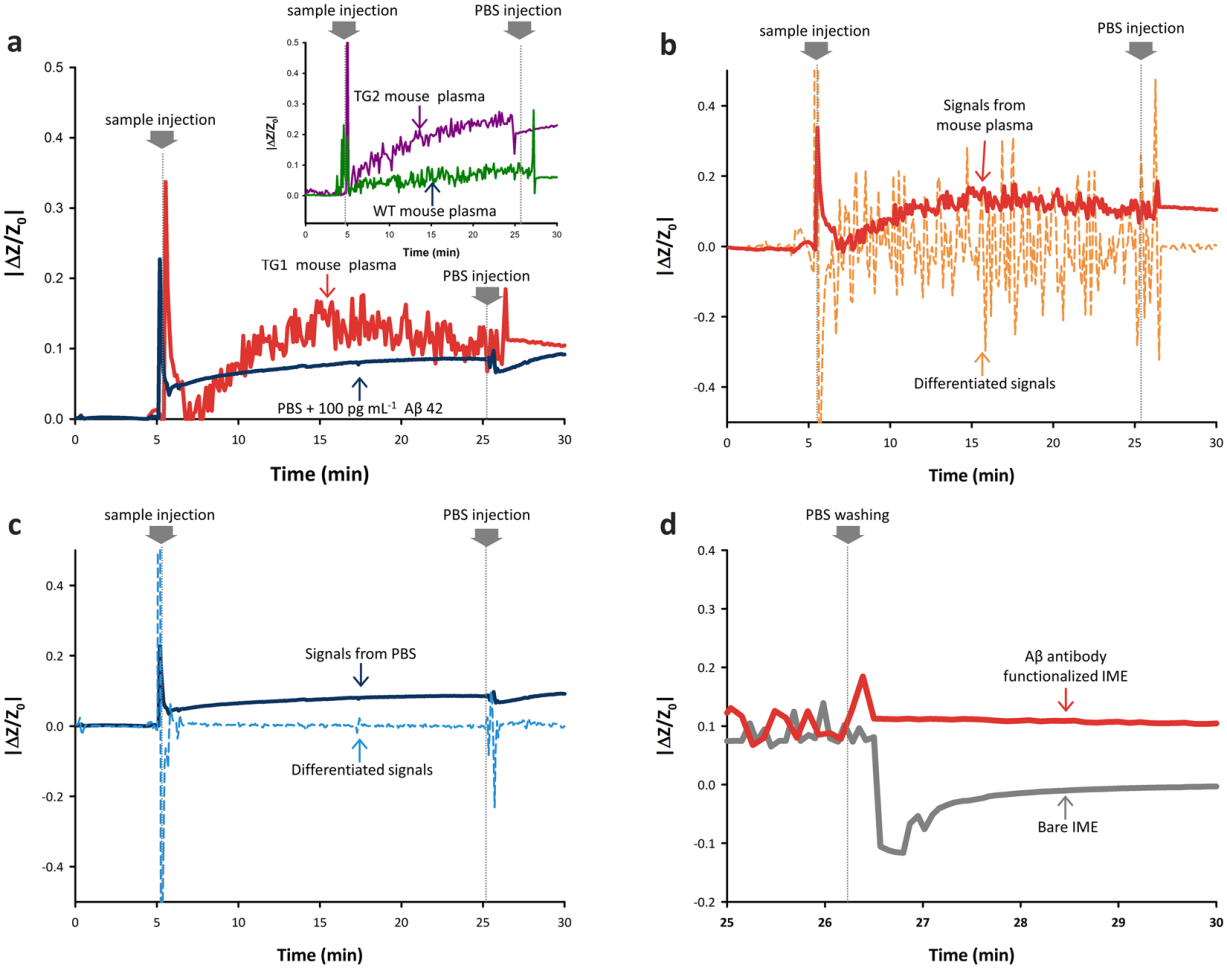
Compatibility test for medium change. (**a**) Real-time detection of Aβ in plasma (TG 1, TG 2 and WT mouse) and PBS. (**b**,**c**) Comparison between signals and differentiated signals from mouse plasma and PBS with Aβ. (**d**) The medium change test for Aβ detection using Aβ-antibody-immobilized and bare IME.

Real-time measurement of impedance changes and comparison with differentiated impedance changes was accomplished to verify the hardness of biomolecule measurement in plasma and confirm the possibility of medium changing measurement after a robust reaction with antibody, as shown in Fig. 4.

As shown in Fig. 4a, 100 pg mL^−1^ synthetic Aβ plus PBS buffer and plasma from the TG (1, 2) and WT mouse were utilized to show the difficulty of Aβ detection in plasma and optimized measurement after medium changes. The TG mouse plasma has almost the same concentration of Aβ as 100 pg mL^−1^ ^11^. Each sample was injected at the PDMS microchannel at 5 min and PBS buffer that had no Aβ was sequentially injected at 25 min for changing the medium. The 5 min before sample injection was set as a stabilization time for the reaction.

During the reaction time from 5 min to 25 min, large fluctuations of unstable signal in impedance changes occurred during measurement with plasma (red (TG 1), violet (TG 2) and green (WT) line in Fig. 4a and inset graph) against PBS buffer (dark blue line in Fig. 4a). Although the reaction leads the tend to increase the impedance with mouse plasma, accurate confinement of the exact impedance changes was not possible due to the fluctuation of signals

For accurate determination of the stable or unstable signal for verification of the hardness of biomolecule measurement in plasma and confirmation of the possibility of medium changing, we defined the conditions as shown in equations (1) and (2).

1 $$|{{\rm{I}}}_{{\rm{m}}}|\ge {\rm{\alpha }}|\frac{{{\rm{dI}}}_{{\rm{m}}}}{{\rm{dt}}}|\,{\rm{or}}\,|{{\rm{I}}}_{{\rm{m}}}|\gg |\frac{{{\rm{dI}}}_{{\rm{m}}}}{{\rm{dt}}}|$$

2 $$\frac{{{\rm{dI}}}_{{\rm{m}}}}{{\rm{dt}}}\propto 0$$

where I_m_ and $\frac{{{\rm{dI}}}_{{\rm{m}}}}{{\rm{dt}}}$ are the changes in impedance ($|\frac{{\rm{\Delta }}Z}{{{\rm{Z}}}_{0}}|\,$in Fig. 4) and differentiated changes of impedance according to time, respectively, and α is the stable coefficient, which was defined as 10 in this experiment. For more accurate determination, α can be set higher than the value. We tried to quantitatively determine the stable signal at which there were much lower $\frac{{{\rm{dI}}}_{{\rm{m}}}}{{\rm{dt}}}$ as compared to the absolute values of I_m_ from equation (1) and almost zero of $\frac{{{\rm{dI}}}_{{\rm{m}}}}{{\rm{dt}}}$ from equation (2).

With the determination of equations for signal stability, verification of measurement hardness in blood was properly tried. The absolute values of I_m_ in each medium shown in Fig. 4a were reputably displayed with the values of $\frac{{{\rm{dI}}}_{{\rm{m}}}}{{\rm{dt}}}$ for plasma and PBS buffer in Fig. 4b and c, respectively. During the reaction time (from 5 min to 25 min) for mouse plasma, the value of $\frac{{{\rm{dI}}}_{{\rm{m}}}}{{\rm{dt}}}$ (dashed line in Fig. 4b) was much higher than the absolute values of I_m_ (red solid line in Fig. 4b). It is not sufficient with determined condition at equation (1) and obviously show that fluctuation of unstable signal disturb the completely accurate acquirement of I_m_ for Aβ in the plasma.

In the case of the PBS buffer, the I_m_ was over 10 times higher than the $\frac{{{\rm{dI}}}_{{\rm{m}}}}{{\rm{dt}}}$ to sufficient the condition from equation (1) as shown in reaction time (from 5 min to 25 min) in the dashed line of Fig. 4c, compared with the measurement in plasma. The value of $\frac{{{\rm{dI}}}_{{\rm{m}}}}{{\rm{dt}}}$ was almost zero in most of reaction time. With the confirmation of stable reaction with PBS buffer, we can understand that the impedance started to increase after the injection at 5 min and was saturated as 12.1% with 100 pg mL^−1^ Aβ at 20 min. The value of stabilized impedance changes after only PBS injection at 25 min was almost the same as the deducted saturation point at 20 min. From this, we can consider that the PBS buffer is sufficiently stable for measurement of the biomolecules in the liquid phase and sufficient to be a replacement medium than plasma.

With the stable measurement with PBS buffer, the media change was also demonstrated with pure PBS buffer. As shown after 25 min for plasma (red line in Fig. 4a and b), the signal had tend to be stable by the medium change with the PBS buffer as Aβ in the PBS buffer.

The medium change method can be produced by the robust reaction with immobilized Aβ antibody, as shown in Fig. 4d. The Aβ-antibody-functionalized IME and bare IME that had no specific antibody for Aβ were utilized. The same reaction was performed with 100 pg mL^−1^ Aβ contained PBS buffer. After only injection of PBS at 25 min, the changes in impedance recovered to 0 values, as indicated by the gray line in Fig. 4d. The value with the Aβ-antibody-functionalized IME, however, was stable (≈12%) after the injection of PBS buffer. This finding shows that the robustly functionalized antibody could react with Aβ in 20 min and medium change could be applicable with plasma base measurement.

In total, all the signals after the medium change to PBS buffer during plasma-based measurement (after 25 min of the solid red line in Fig. 4a and b) showed uniformly saturated and stable values of approximately 13%. This is quite close to the value obtained due to the changes caused by PBS buffer containing 100 pg mL^−1^ of Aβ after 25 min, as shown by black solid line in Fig. 4a.

The results of these verifications and analyses indicated that the medium-changing method could aid in detection of the low concentration of Aβ in plasma with the IME sensor system.

### Aβ detection in mouse plasma samples

**Figure 5:**
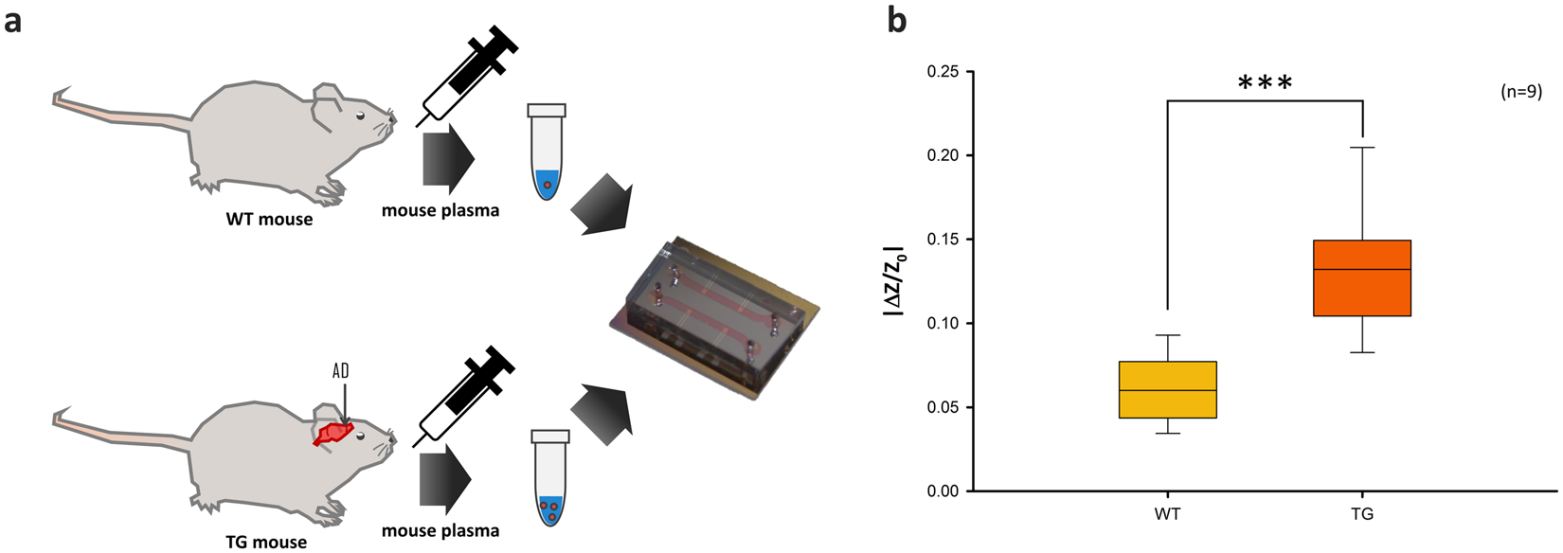
Mouse model test. (**a**) Scheme for mouse plasma collection and detection. (**b**) Mouse plasma sample test for discrimination between AD and healthy controls (n = 9, ***significant at p < 0.001).

Based on the highly sensitive verification of selective responses to Aβ, animals were tested using mouse plasma samples with SCAP system (Fig. 5b). Plasma samples from APP/PS1 transgenic (TG, n = 9) and wild-type (WT, n = 9) 7- and 8-month-old female mice were collected for nine evaluations. Transgenic mice were modified to develop AD. Each plasma sample was injected into IMEs and impedance changes of IMEs were monitored. The median of increase in impedance of wild-type mouse plasma sample–injected IMEs was approximately 5.9% (Fig. 5b). The upper quartile and lower quartile were 7.5% and 4.3%, respectively. The median was calculated to be dozens of picograms per milliliter Aβ of the normal control (WT). The median increase in impedance for TG mouse plasma sample–injected IMEs was approximately 13.1%, which was calculated to be several hundred picograms per milliliter Aβ in AD patients; 14.7% of the upper quartile and 10.4% of the lower quartile were measured. Thus, this result demonstrates that physiological samples used for AD diagnosis can be discriminated using the cancellation process (Fig. 5b). We expect high sensitivity and resolution for this biosensor system in the range of 100 fg mL^−1^ to 1 ng mL^−1^ Aβ in physiological samples.

### Comparative discussion of different sensing platform for blood plasma based-Aβ detection

Previously, various type sensing platform have been developed for Aβ detection. Many research group was investigated that dynamic range of Aβ sensing platform coincide in the clinically meaningful range for blood-based AD diagnosis^7, 32^. For the simplicity of detection process, various sensing platform are investigated with label-free^7, 10, 14, 33–36^. And, for the complex sample detection, blood based Aβ detection have been investigated by several research group^10, 33, 37^. Compare to previous research, our Aβ sensing platform for AD diagnosis has several advantages as below. The dynamic range (0.61 pg/mL to 1 ng/mL) of IME is closely coincide in the clinically meaningful range. In the dynamic range, the linear relation of sensitivity of 0.07679 (|∆Z/Z_0_|)/(pg mL^−1^) was estimated. Also the medium change method was considered for the blood based-Aβ detection. In this study, we expect that an IME for Aβ detection could provide the possibility of blood-based AD diagnosis with high sensitivity. The compared previous studies and this work are described in supplementary information (Table S2).

## Conclusions

In summary, we developed an optimized sensor platform for blood-based AD diagnosis by using an IME with SCAP system and medium changes. The IME chip with a PDMS microchannel was demonstrated for detection of Aβ. The additional measurement system with cancellation and amplification processes was also utilized to enhance sensitivity. Optimization of medium change with PBS buffer and verification of hardness in plasma-based detection of Aβ at low concentration were also accomplished. With the derived sensor platform and sample preparation method, we performed measurements of Aβ in wild-type and TG mouse plasma samples, which confirmed the possibility of diagnosing AD using this method for detection of Aβ levels with high sensitivity and resolution. We anticipate that this sensor platform could be used for blood-based AD evaluation with low costs, a simple procedure, and high efficiency. Furthermore, an IME with a sensing system could considerably improve the sensitivity and resolution of biosensors and chemical sensor platforms.

## Electronic supplementary material

Supplementary Information
