# Supplementary material for: A highly sensitive plasma-based amyloid-β detection system through medium-changing and noise cancellation system for early diagnosis of the Alzheimer’s disease
Source: Sci Rep. 2017 Aug 21;7:8882. doi: 10.1038/s41598-017-09370-3 (PMC5567090; doi:10.1038/s41598-017-09370-3)
Supplement: Supplementary file 1 — Supplementary Information [file 41598_2017_9370_MOESM1_ESM.pdf]

A highly sensitive plasma-based amyloid-β detection system through medium-changing and noise cancellation system for early diagnosis of the Alzheimer's disease

Yong Kyoung Yoo[1,2*], Jinsik Kim[3*], Gangeun Kim[4], YoungSoo Kim[5], Hye Yun Kim[5], Sejin Lee[5], Won Woo Cho[6], Seongsoo Kim[7], Sang-Myung Lee[7], Byung Chul Lee[4], Jeong Hoon Lee[2], Kyo Seon Hwang[1 †]

[1]Department of Clinical Pharmacology and Therapeutics, College of Medicine, Kyung Hee University, Seoul 02447, Korea

[2]Department of Electrical Engineering, Kwangwoon University, Seoul 139-701, South Korea

[3]Department of Medical Biotechnology, College of Life Science and Biotechnology, Dongguk University, Seoul, Korea

[4]Center for BioMicrosystems, Korea Institute of Science and Technology (KIST), Seoul 136-791, South Korea.

[5]Department of Pharmacy, Yonsei University, Incheon 21983, South Korea

[6]CANTIS.co, Sangnok-gu, Ansan-si, Gyeonggi-do 426-901, South Korea

[7]Department of Chemical Engineering, Kangwon National University, Gangwon-do 200-701, South Korea

[†] Corresponding author at: Department of Clinical Pharmacology, College of Medicine, Kyung Hee University, Seoul 02447, Korea

E-mail address: k.hwang@khu.ac.kr (K.S. Hwang).

[*] These authors contributed equally.

a

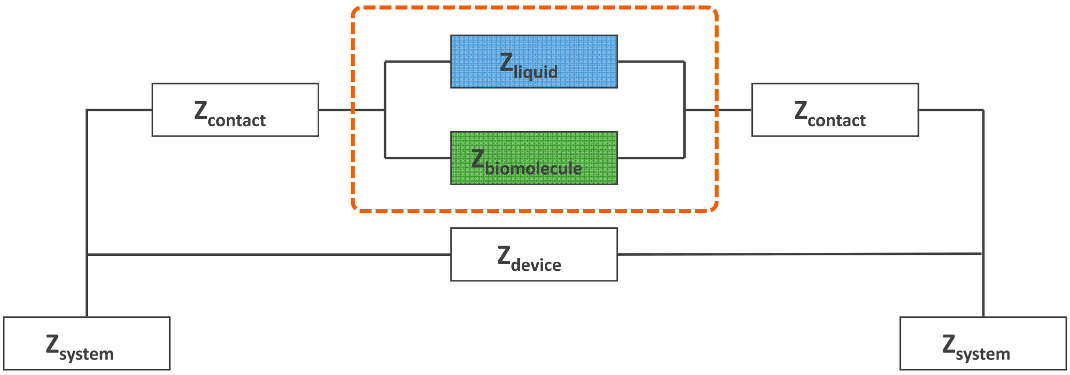

b

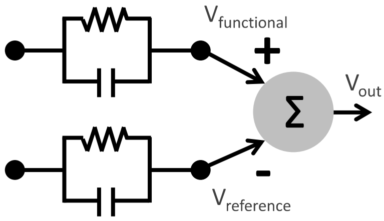

Fig. S1. Equivalent circuit of IME sensor and cancellation principle.

The designed interdigitated microelectrode was used for Aβ protein detection with signal processing systems. The interaction between Aβ protein and Aβ antibody, which is immobilized at the functional electrode of the sensor, led to changes in the impedance. Fig. S1. (a) shows an equivalent circuit of IME sensor in buffer solution before the functional electrode for accurate measurement of impedance changes by the interaction between Aβ protein and Aβ antibody ($Z_{biomolecule}$). The biomolecule-containing liquid has high impedance. The designed

system was utilized to cancel out the impedance of liquid (Z_{liquid}) and the specified parasitic impedance caused by the system and device. The Z_{system} and $Z_{contact,}$ which respectively represent impedance due to the measurement system and measuring probe and electrode, were considered in the equivalent circuit. The Z_{device} represents the impedance between electrodes in a single device. As shown in Fig. S1, the reference electrode was used to cancel out these all impedances for enhancement of sensitivity. As shown in Fig. S1(b), the voltages from the functional electrode ($V_{functional}$) and reference electrode ($V_{reference}$) were calculated to cancel out other impedances for the accurate measurement of impedance changes by only the $Z_{biomolecule}$ with the system. The output voltage (V_{out}), which is due to the $Z_{biomolecule}$, was also amplified with the amplifier after the cancellation between the voltages of functional and reference electrodes as differential amplifier works.

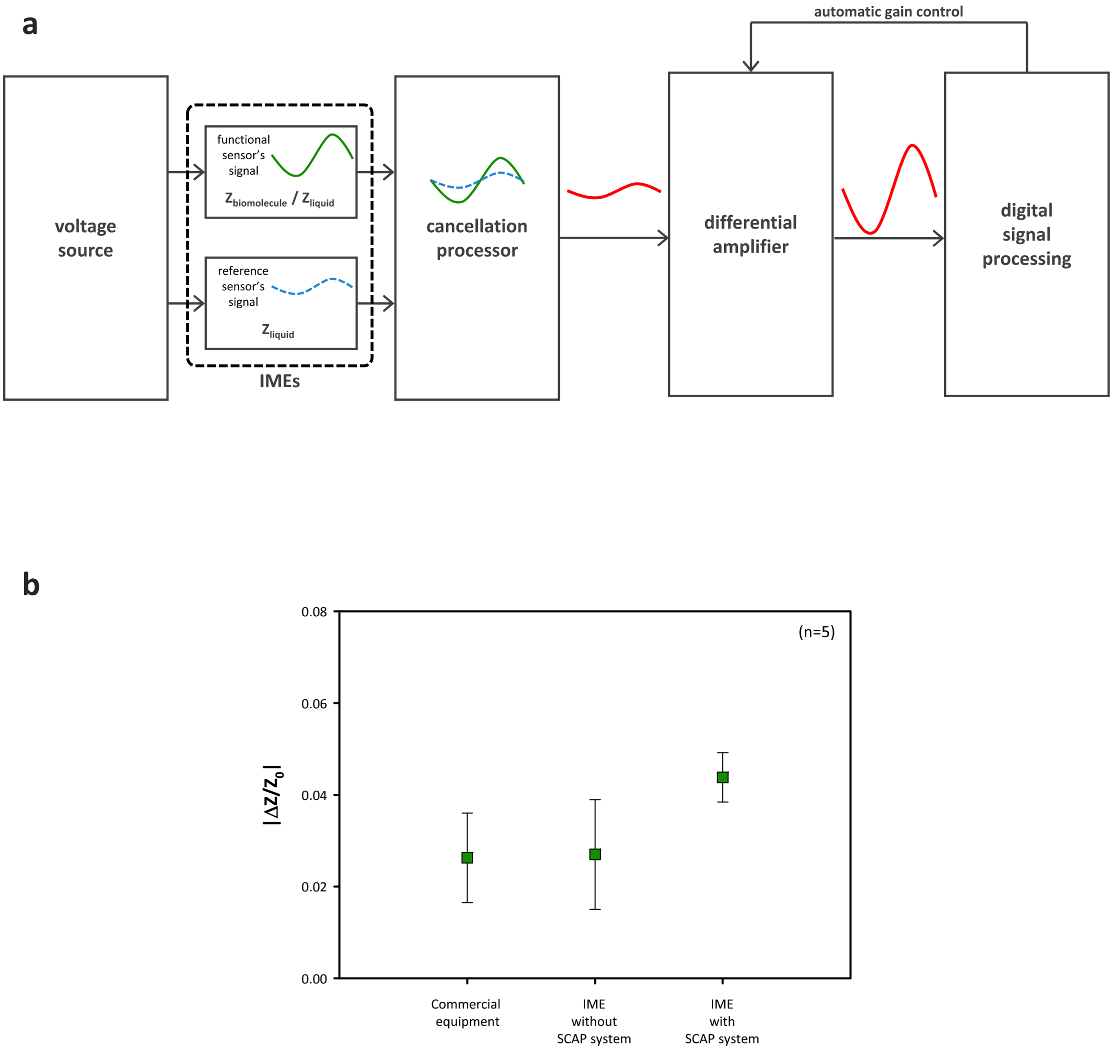

Fig. S2. Performance of signal cancellation and amplification process system embedded measurement system.

After obtaining an Aβ antibody layer on SiO_2, the interaction between the immobilized Aβ antibody and Aβ antigen could be measured using the designed IME sensing system. The designed sensing system, which has a signal cancellation and amplification process (SCAP) system embedded in the measurement system, was accomplished as shown in Fig. S2(a) in order to enhance sensitivity along with noise removal. Generally, the noise occurred by a biomolecule-containing solution and the structure of IME, which has a relatively long electrode length between electrodes. The Z_{liquid} and $Z_{biomolecule}$ marked in Fig. S2(a) indicate the impedance of solution, including the effect of IME structures and impedance of target biomolecules,

respectively. The Z_{liquid} and $Z_{biomolecule}$ were more dominant than other impedance components, as shown in an equivalent circuit (see Fig. S1(a) for detatils.). The effect of Z_{liquid} was approximately 1000–2000 fold greater than that of $Z_{biomolecule}$. Therefore, we developed a cancellation and amplification process to remove Z_{liquid} and other impedance components. Functional and reference electrodes were utilized. The functional electrode could detect $Z_{biomolecule}$, including the effect of liquid, Z_{liquid}. The reference electrode only detected Z_{liquid}. Therefore, we loaded the biomolecule-containing solution at the functional electrode and the same liquid without biomolecules at the reference electrode. The same conditions, voltage, and frequency were applied at both electrodes. After measurement of two impedimetric signals by the functional and reference electrodes, the two signals ($Z_{biomolecule}/Z_{liquid}$ and Z_{liquid}) were subtracted, to obtain the changes that were due to biomolecules ($Z_{biomolecule}$). The amplification was also applied after the cancellation. Finally, we acquired amplified signals obtained only due to biomolecules ($Z_{biomolecule}$) with the designed measurement systems. Other modes that do not apply cancellation and amplification could also be used.

To verify IME impedance measuring system, we repeatedly compared SCAP system with commercial equipment (PGSTAT302N, Metrohm Autolab), as shown in Fig. S2(b). After 10 pg mL-1 Aβ–Aβ antibody interaction, the impedance change of the same IME was monitored using each measurement system. About 2.6%, 2.7%, and 4.3% of impedance changes were measured using commercial equipment, IME without SCAP system, and IME with SCAP system, respectively; approximately 1.9%, 2%, and 1% standard deviations, respectively, were calculated for the impedance changes. By utilizing IME with SCAP system, we achieved a 1.6-fold higher impedance change and a half lower standard deviation compared with other systems. We also confirmed that the IME with SCAP system for Aβ detection was more suitable than the other systems.

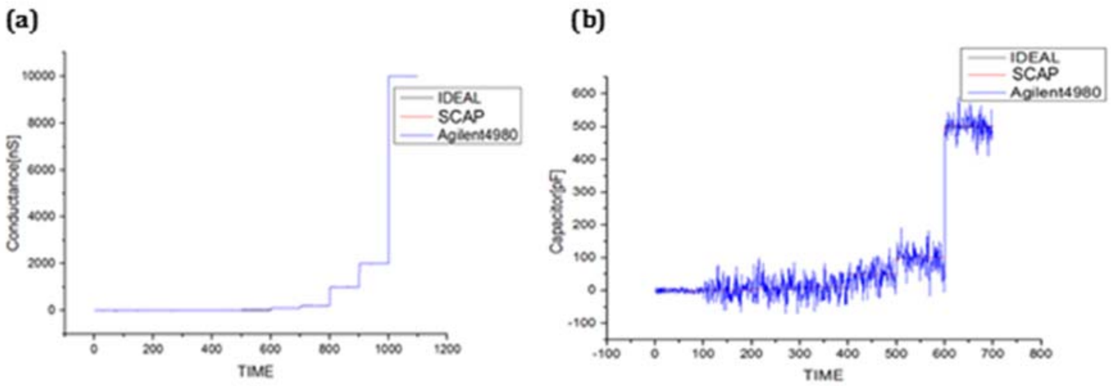

Fig. S3. (a) Conductance and (b) capacitance measurement with SCAP and commercial equipment (Agilent 4980).

Table. The error ratio estimation of measuring conductance and capacitance

**Capacitance (pF)**	**Error ratio (%)**	
	**SCAP**	**commercial**
**10**	0.59	64.908
**50**	0.15	1.293
**100**	0.47	4.361
**500**	0.38	0.177
**average**	0.39	17.68

**Resistance (MΩ)**	**Error ratio (%)**	
	**SCAP**	**commercial**
**200**	9.47	31.89
**100**	5.06	13.94
**50**	10.14	15.89
**10**	2.52	4.06
**5**	0.39	1.37
**1**	0.29	0.07
**0.5**	0.26	0.09
**0.1**	0.08	0.05
**average**	3.52	8.42

Before the application for IME sensor, the impedance measuring system with SCAP is evaluated. First, we prepared and compounded the electrical components (resistor of 0.1, 0.5, 1, 5, 10, 50, 100, 200 MΩ and capacitor of 10, 50, 100, 500 pF). The conductance and capacitance were monitored with SCAP and commercial equipment (Agilent 4980) as shown Fig. S3. When the conductance and the capacitance measured, the ratio of error of conductance and capacitance with SCAP is reduced as described in Table. Furthermore, IME's resistance of approximately 0.4 MΩ and capacitance of 170 pF were measured, respectively. In the capacitance of range 100 to 500 pF and resistance of range 0.1 to 0.5 MΩ, the impedance measuring system with SCAP is more appropriate with low measuring error.

Table S1. Biosensors for the detection of Aβ

Reference

**Sensing platform**	**Simplicity**	**Detection range**	**Real bio-fluidics were utilized?**	**Reference**
rGO-FET	Label-free	1 fM to 100 pM	No	1
CNT-MESFET	Label-free	1 pg/mL to 1 ng/mL	Synthetic analytes in human serum	2
GO-based fluorescence	fluorescence label	10 nM to 2 mM	No	3
QCM	Label-free	50 pg/mL to 5 μg/mL	No	4
DVP	labeled	100 pM to 50 nM	Synthetic Aβ in CSF of both normal and AD rats	5
Cyclic voltammetry	fluorescence label	20 pM to 1.50 nM	Synthetic Aβ added into artificial CSF	6
Cyclic voltammetry with N-doped graphene modified Au electrodes	FITC label	5 pg/mL to 800 pg/mL	No	7
SERS	Labe-free	500 fg/mL to 6 pg/mL	Synthetic Aβ spiked in rabbit whole blood.	8
EIS	Label-free	1 pM to 1 μM	Cell-derived natural Aβ oligomers in culture medium	9
EIS with carbon disposable electrochemical printed chip	Label-free	10 pM to 200 μM	No	10
**This work**	**Label-free**	**100 fg/mL to 1 ng/mL (synthetic Aβ)**	**Synthetic Aβ in mouse plasma**	

1 Kurkina, T. *et al.* Self-Assembled Electrical Biodetector Based on Reduced Graphene Oxide. *ACS Nano* **6**, 5514-5520, (2012).

2 Oh, J. *et al.* A carbon nanotube metal semiconductor field effect transistor-based biosensor for detection of amyloid-beta in human serum. *Biosensors and Bioelectronics* **50**, 345-350, (2013).

3 Liu, L. *et al.* A graphene oxide-based fluorescent platform for selective detection of amyloid- β oligomers. *Analytical Methods* **7**, 8727-8732, (2015).

4 Mustafa, M. K. *et al.* Detection of β-amyloid peptide (1–16) and amyloid precursor protein (APP770) using spectroscopic ellipsometry and QCM techniques: A step forward towards Alzheimers disease diagnostics. *Biosensors and Bioelectronics* **26**, 1332-1336, (2010).

5 Yu, Y. *et al.* Gelsolin bound β-amyloid peptides(1–40/1–42): Electrochemical evaluation of levels of soluble peptide associated with Alzheimer's disease. *Biosensors and Bioelectronics* **68**, 115-121, (2015).

6 Liu, L. *et al.* Electrochemical detection of β-amyloid peptides on electrode covered with N-terminus-specific antibody based on electrocatalytic O2 reduction by Aβ(1–16)-heme-modified gold nanoparticles. *Biosensors and Bioelectronics* **49**, 231-235, (2013).

7 Li, S.-S. *et al.* Non-invasive screening for early Alzheimer's disease diagnosis by a sensitively immunomagnetic biosensor. *Scientific Reports* **6**, 25155, (2016).

8 Demeritte, T. *et al.* Hybrid Graphene Oxide Based Plasmonic-Magnetic Multifunctional Nanoplatform for Selective Separation and Label-Free Identification of Alzheimer's Disease Biomarkers. *ACS Applied Materials & Interfaces* **7**, 13693-13700, (2015).

9 Rushworth, J. V. *et al.* A label-free electrical impedimetric biosensor for the specific detection of Alzheimer's amyloid-beta oligomers. *Biosensors and Bioelectronics* **56**, 83-90, (2014).

10 Lien, T. T. N., Takamura, Y., Tamiya, E. & Vestergaard, M. d. C. Modified screen printed electrode for development of a highly sensitive label-free impedimetric immunosensor to detect amyloid beta peptides. *Analytica Chimica Acta* **892**, 69-76, (2015).
